# Mechanical Comparison of Biofilms with Altered Matrix Composition: A Study Combining Shear-Rheology and Microindentation

**DOI:** 10.1021/acsbiomaterials.5c00261

**Published:** 2025-06-12

**Authors:** Macarena Siri, Adrien Sarlet, Ricardo Ziege, Laura Zorzetto, Carolina Sotelo Guzman, Shahrouz Amini, Regine Hengge, Kerstin G. Blank, Cécile M. Bidan

**Affiliations:** † Max Planck Institute of Colloids and Interfaces, 14476 Potsdam, Germany; ‡ Humboldt University, 10115 Berlin, Germany; § Johannes Kepler University Linz, Institute of Experimental Physics, Altenberger Str. 69, 4040 Linz, Austria

**Keywords:** E. coli, biofilm, mechanical properties, extracellular matrix

## Abstract

The mechanical properties of bacterial biofilms depend on the composition and microstructure of their extracellular matrix (ECM), which constitutes a network of extracellular proteins and polysaccharide fibers. In particular, macrocolony biofilms were suggested to present tissue-like elasticity due to a dense fiber network consisting of amyloid curli and phosphoethanolamine-modified cellulose (pEtN-cellulose). To understand the contribution of these two main ECM components to the emergent mechanical properties of biofilms, we performed shear-rheology and microindentation experiments on biofilms grown from strains that produce different ECM. We measured that biofilms containing curli fibers are stiffer in compression than curli-deficient biofilms. We further quantitatively demonstrate the crucial contribution of pEtN-cellulose, and especially of the pEtN modification, to the stiffness and structural stability of biofilms when associated with curli fibers. To compare the differences observed between the two methods, we also investigated how the structure and mechanical properties of biofilms with different ECM compositions are affected by the sample preparation method used for shear-rheology. We found that biofilm homogenization, used prior to shear-rheology, destroys the macroscale structure of the biofilm while the microscopic ECM architecture may remain intact. The resulting changes in biofilm mechanical properties highlight the respective advantages and limitations of the two complementary mechanical characterization techniques in the context of biofilm research. As such, our work does not only describe the role of the ECM on the mechanical properties of biofilms. It also informs the biofilm community on considering sample preparation when interpreting mechanical data of biofilm-based materials.

## Graphical abstract

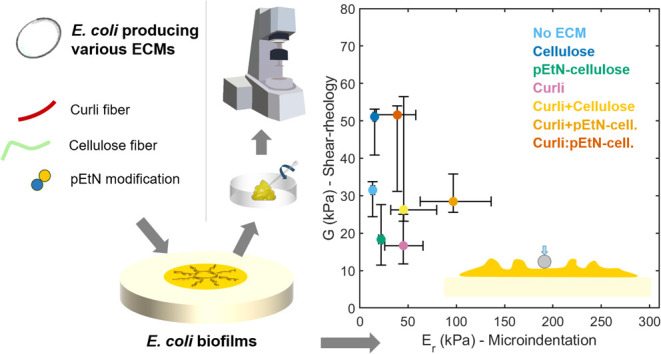

## Introduction

Biofilms are complex, highly heterogeneous living materials, composed of bacteria embedded in a self-produced extra­cellular matrix (ECM) of proteins and polysaccharides. In recent decades, biofilm mechanics has been studied across diverse fields, from microbiology to materials science and soft matter physics.^1^ In the medical field, disrupting biofilm cohesion is a standard procedure in chronic wound care, making the remaining adherent bacteria more susceptible to antibiotics.^2^ Understanding biofilm mechanics is also essential for preventing biofilm formation or for leveraging their benefits in industrial and bioprocessing applications, such as wastewater treatment.^3^ Broadly, research on biofilm mechanics aims to deepen our understanding of the biofilm life cycle,^4^ clarify how the viscoelastic properties of biofilms aid bacteria survival in mechanically challenging environments (e.g., under flow)^5^ and inspire new approaches for the mechanical removal of biofilms.^6^

Biofilms are often described as complex fluids with viscoelastic material properties^7^ or as composite materials, where relatively rigid colloid-like bacteria are embedded within a soft, hydrated ECM composed of various biopolymers.^8^ The ECM accounts for a significant volume fraction of the dry biomass so that the nature of the biopolymers, their molecular structure as well as their interactions are expected to govern the biological material responses at macroscopic scales.^9,10^ The elastic modulus (also called Young’s modulus or stiffness) is a mechanical parameter broadly used to report on the rigidity of a material, e.g., the elastic modulus of bone is about 20 GPa and 1000 times higher compared to skin (20 MPa). In cystic fibrosis, the bacteria that colonize lungs increase biofilm elastic modulus by increasing their production of the ECM polysaccharide Psl, whereas an increased expression of Pel and alginate has no influence on the biofilm stiffness.^11^ Similarly, biofilms become more ductile (less brittle) when the ECM-to-cell ratio is increased.^12^ Recently, it was found that the molecular structure of curli amyloid fibers extracted from biofilms depends on the growth conditions and correlates with biofilm stiffness.^13^ Interactions between the ECM components produced by were also shown to promote biofilm cohesion and prevent mechanical biofilm failure when scraping with the tip of an indenter.^14^

Biofilm structural heterogeneity contributes to its mechanical complexity. Gradients of nutrients and oxygen, emerging across the biofilm, result in gradients of metabolic activities and ECM production.^7^ In the macrocolony model, for example, cross sections of biofilms, grown on the surface of nutritive agar plates, revealed layers with distinct ECM content and organization.^15^ The mechanical stability of such AR3110 macrocolony biofilms was proposed to benefit from the assembly of a curli and phosphoethanolamine-cellulose (pEtN-cellulose) fiber network, which provides tissue-like elasticity.^16,17^ The small number of studies reporting on the mechanical properties of biofilms did, however, not specifically assess the mechanical implications of such an elaborate architecture.^18^ Macrocolony biofilms from various species were also shown to exhibit porous structures or channels for nutrient transport and diffusion.^19–21^ It is expected that local composition and architecture determine biofilm mechanics at the microscopic scale. As a result, the mechanical properties are highly heterogeneous throughout the biofilm and differ significantly from the macroscopic properties.^22^ Due to the living nature of biofilms, their mechanical properties further depend on the time scale considered (e.g., short time scales accounting for ECM rearrangement or long time scales related to growth phenomena or changes in gene expression).

To characterize the viscoelastic properties of macrocolony biofilms, the choice of method as well as the accessible time and length scales are essential aspects to consider.^1^ Oscillatory shear-rheology has been widely used to assess the bulk viscoelastic properties of bacterial macrocolony biofilms produced by .,^23^ ,^24^ and .^25^ While this method gives information at the macroscopic scale, the sample preparation often involves removing the biofilms from their substrate. This may be possible with cohesive biofilms such as those from ,^26^ but is potentially destroying ECM architecture in other cases.^27^ An alternative is to grow biofilms on a semipermeable membrane placed on the agar substrate, allowing for a nondestructive transfer to the rheometer plate.^28^ However, such a substrate may affect biofilm morphogenesis. Another strategy consists of growing biofilms directly on the rheometer sample stage, as demonstrated with several strains of and .^29^ For measuring local properties of native macrocolony biofilms, atomic force microscopy (AFM) is attractive as it provides height and stiffness maps with single-cell spatial resolution.^30^ Microindentation may be better suited for microscale mechanical characterization (as opposed to nanoscale). On the macrocolony scale (centimeter-sized), indenters with a diameter of tens of micrometers provide local information while still averaging over the contribution of several tens of bacteria and the surrounding fibrous ECM network. For example, microindentation showed that growing on drier agar substrates yields stiffer biofilms.^31^ Modeling stress relaxation of biofilms in rheology experiments revealed multiple characteristic time scales as a function of ECM composition.^32^ Local biofilm viscoelastic properties can also be obtained from passive or active microrheology, i.e., by tracking the Brownian motion or induced displacement (with magnetic or optical tweezers) of microparticles embedded in the ECM.^27^ This last approach is preferred for immersed biofilms grown in liquid, e.g., in flow chambers.^33,34^ Overall, the diversity of mechanical tests available, the lack of standardization in the protocols for biofilm characterization and the complex response of biofilms to deformations lead to difficulties in comparing mechanical parameters across different studies,^27^ even when considering the same bacterial strain grown in different^1^ or very similar conditions.^35^

In this work, we investigated the contributions of the main ECM components of biofilms, namely curli amyloid and pEtN-cellulose fibers, to the mechanical properties of the macrocolony biofilm material. Moreover, we explored the relevance of their interactions as well as the integrity of their architecture for the viscoelastic behavior of the biofilm. We grew different K-12 mutants on nutritive agar substrates to obtain macrocolony biofilms of different ECM compositions, and characterized their viscoelastic properties using oscillatory shear-rheology and microindentation. For rheology, the biofilm material was scraped from the agar substrate and homogenized while the native biofilm architecture was preserved when performing microindentation. Comparing the two characterization methods reflects the difficulties mentioned above.^27^ At the same time, the results indicate that curli amyloid fibers play a key role in determining biofilm rigidity while cellulose, especially pEtN-cellulose, contributes to maintaining its structural integrity. Further structural investigations using confocal microscopy suggest that the composite nature and structural heterogeneity of the macrocolony biofilms make them sensitive to the specific methods used for mechanical characterization. Notably, this sensitivity is influenced by the composition of the ECM. All in all, this work does not only bridge an important gap in the mechanical characterization of macrocolony biofilms. It also reminds the biofilm community that multimodal mechanical characterization is needed to understand the mechanical properties of biofilm-based materials.

## Results

### Influence of EPS Synthesis on Macrocolony Biofilm Morphology, Texture, and Mass

To investigate the influence of the ECM composition on biofilm growth and material properties, we cultured wild-type and mutant strains with varying capacities to produce ECM components for 7 days on salt-free LB agar substrates. The different strains produce neither curli nor cellulose (AR198, also referred to as “No ECM”), only nonmodified cellulose (AP472), only pEtN-cellulose (AP329), only curli (W3110), curli and nonmodified cellulose (AP470), and finally both curli and pEtN-cellulose fibers (AR3110). Moreover, we grew biofilms from a bacterial suspension containing a 1:1 mixture of W3110 and AP329 so that both curli and pEtN-cellulose should be present but produced by different bacteria (Figure 1A).

**Figure 1:**
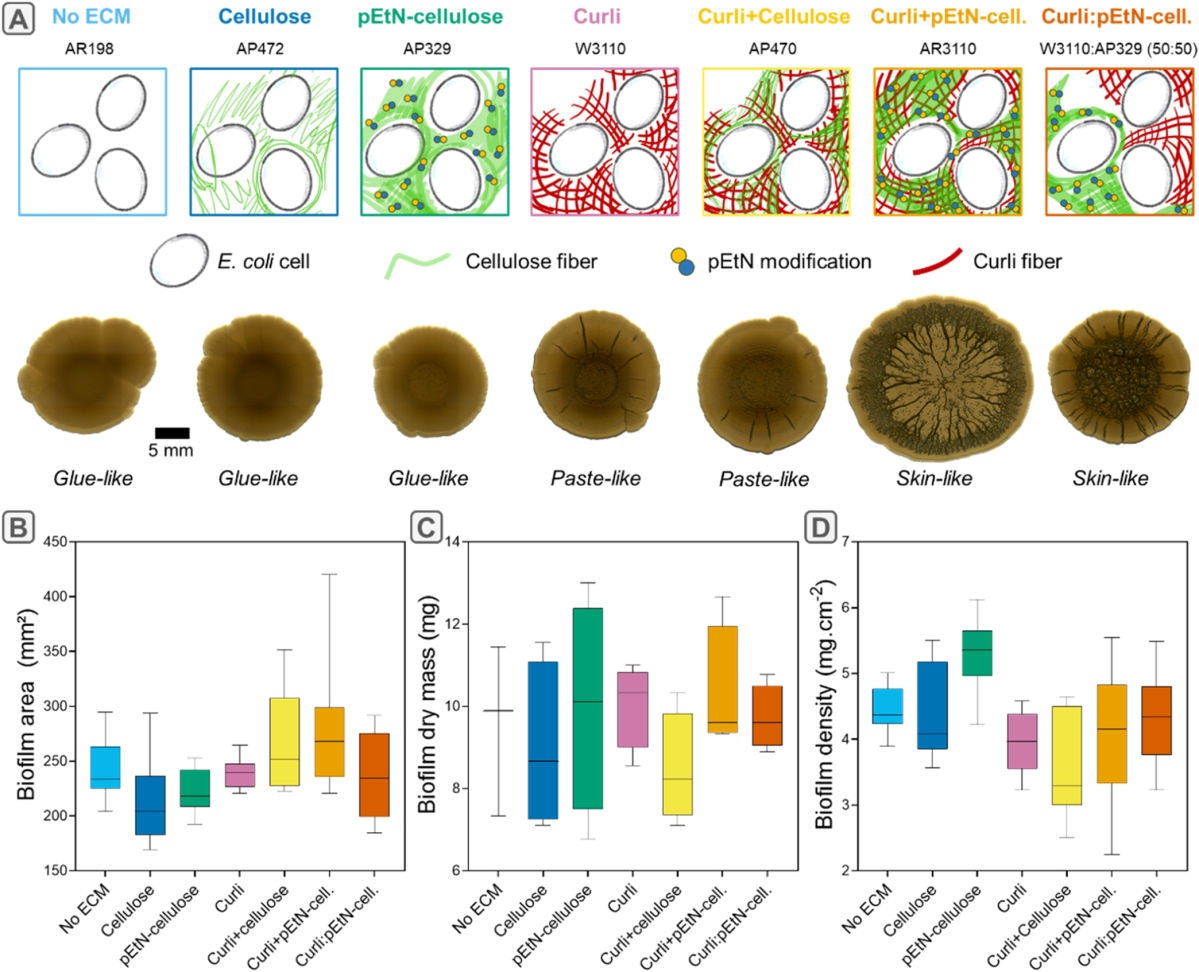
ECM composition influences macrocolony biofilm spreading, texture, dry mass and water content. (A) Morphologies and textures of biofilms grown from strains with various ECM compositions (Table S1). The sketches at the bacteria scale are based on existing knowledge on the respective strains.^16,36^ The images were acquired after 7 days of growth using a stereomicroscope in transmission. The darker lines result from the emergence of wrinkles in the third dimension. Textures are qualitative descriptions based on biofilm properties when scraped from the agar surface. (B–D) Biofilm diameter (*n* = 12 biofilms produced in 4 different plates), dry mass (*N* = 3–4 plates per strain) and density as a function of composition; median, quartiles and extreme values are represented by the line, the box limits and the whiskers respectively; Mann–Whitney U-tests were performed for statistical analyses. Statistical significance values are reported in Tables S2–4.

As reported before, ECM composition greatly influences macrocolony biofilm morphology and texture.^36,37^ We observed that biofilms grown from curli-producing bacteria present apparent wrinkles, while others do not. Biofilms containing both curli and pEtN-cellulose form numerous and predominantly radial delaminated wrinkles independent of whether these fibers are produced by the same (AR3110) or different bacteria (W3110:AP329). Under our experimental conditions, only these two cases yielded biofilms with a skin-like texture upon handling, which demonstrates some cohesion in the material. In contrast, biofilms containing only curli fibers (W3110) or curli fibers and nonmodified cellulose (AP470) formed fewer and shallower radial wrinkles. They exhibited paste-like properties, quickly losing the film aspect upon manipulation, while retaining some cohesion (Figure 1A). Biofilms lacking the curli component, i.e., producing only pEtN-cellulose (AP329), only nonmodified cellulose (AP472) or none of the ECM fibers (AR198), all appear featureless and exhibit glue-like textures. These biofilms adhered strongly to the spatula when scraped from the soft agar substrate.

After 7 days of growth, biofilms containing both curli and pEtN-cellulose (AR3110) were slightly more spread out than their counterparts (Figure 1B and Table S2) while biofilms containing only nonmodified cellulose spread slightly less. However, all seem to contain similar amounts of dry mass, i.e., between 7 and 11 mg, with slight but statistically nonsignificant variations from strain to strain (Figure 1C and Table S3). This yields different biofilm surface densities (in mg cm^–1^), accounting for how much biofilm dry mass has formed on a specific area of the substrate (Figure 1D). Even though no apparent macroscopic wrinkles were present, biofilms containing only pEtN-cellulose showed a significantly higher surface density than the other strains (Table S4). Water content and water activity, measured in 7 day old biofilms, did not show statistical differences between strains (Figure S1). It is yet important to highlight that the low masses measured introduce measurement errors that cannot be easily offset by increasing the number of technical repeats across all conditions.

### Influence of ECM Composition on the Bulk Viscoelastic Properties of Homogenized Biofilms Measured by Oscillatory Shear-Rheology

To probe the viscoelastic properties of biofilms with different ECM composition, we performed bulk oscillatory shear-rheology using so-called “homogenized biofilms”. For each strain, a few biofilms grown in the same Petri dish were scraped off the surface and mixed together to collect sufficient material to fill the 250 μm gap between the two parallel plates in the rheometer setup (Figure 2A). Amplitude sweeps were performed to determine the viscoelastic response of the homogenized biofilms to the applied shear strain (Figures 2B and S2). For each ECM composition, the plateau values of the storage modulus (*G*′_0_) were almost 1 order of magnitude larger than those of the loss modulus (*G*″_0_) and the respective differences between the ECM compositions displayed the same trends for *G*′_0_ and *G*″_0_ as indicated by similar tan δ_0_ values (Figure S3A). Comparing the median values of *G*′_0_ and *G*″_0_ for the different strains revealed different viscoelastic responses (Figure 2C,D). For example, homogenized biofilms containing only pEtN-cellulose or curli fibers presented storage moduli of 18 and 16 kPa, respectively. In contrast, biofilms containing both ECM components were stiffer, namely 28 kPa when pEtN-cellulose and curli were produced by the same bacteria (AR3110; Curli + pEtN-cellulose) and 51 kPa when they were synthesized from the mixture of W3110 and AP329, which each produce one type of fiber (Curli:pEtN-cellulose). Interestingly, homogenized biofilms containing only nonmodified cellulose also presented a storage modulus of 51 kPa and biofilms producing neither fiber were not the softest (*G*′_0_ = 31 kPa).

**Figure 2:**
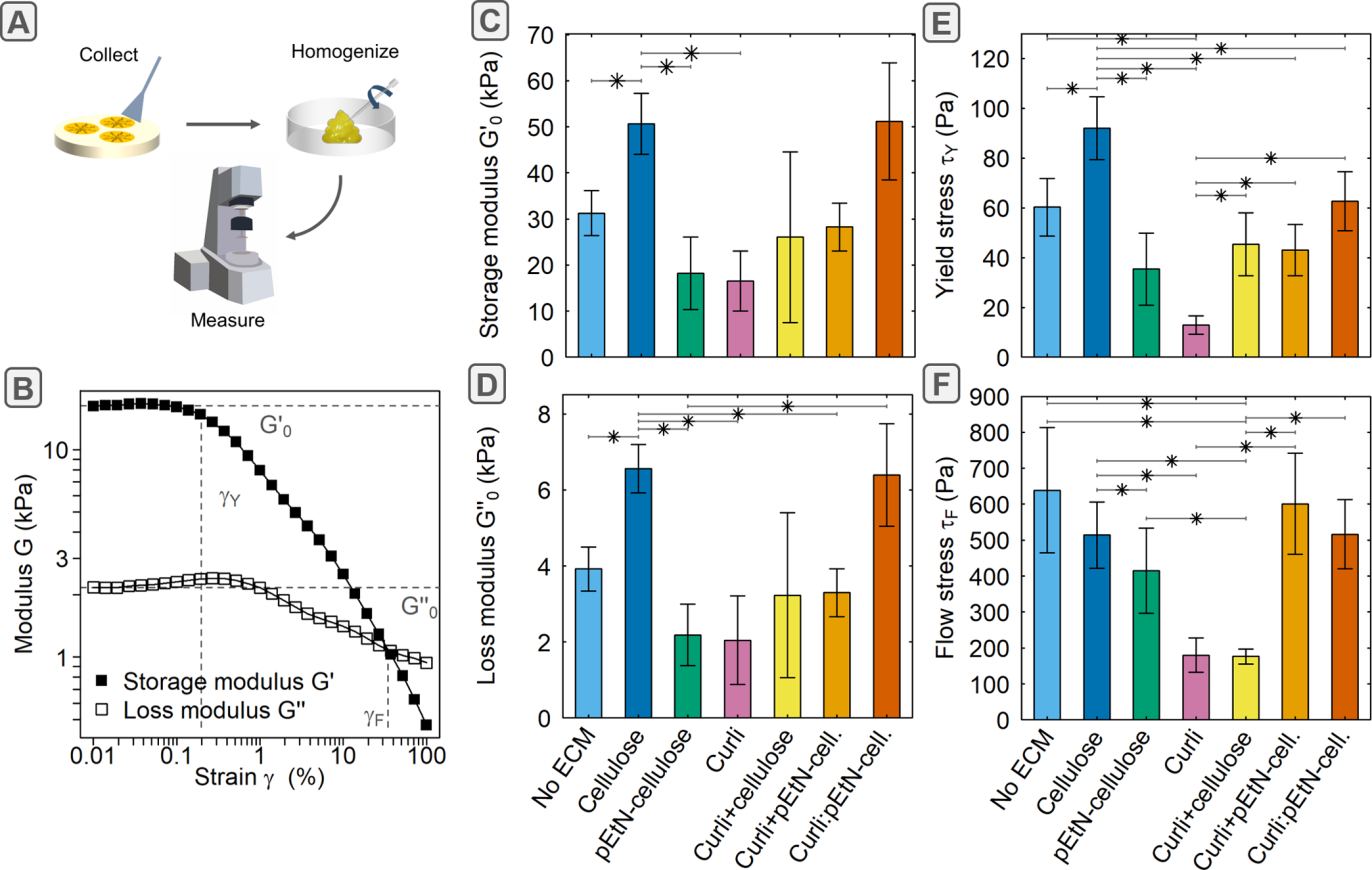
Oscillatory shear-rheology of biofilms with different ECM compositions. (A) Schematic image of sample preparation steps for bulk rheology measurements of homogenized biofilms. (B) Representative strain amplitude sweep (here for AR3110, Figure S2) used to extract the viscoelastic properties of homogenized biofilms (ω = 10 rad s^–1^, γ = 0.01 to 100%). (C) Plateau of the storage modulus (*G*′_0_). (D) Plateau of the loss modulus (*G*″_0_). (E) Yield stress (τ_Y_). (F) Flow stress (τ_F_). (C–F) Bars represent median values and error bars represent standard deviation of bootstrapped data sets. A Mann–Whitney U test was used (*: *p*-value = 0.1, *n* = 3 measurements with biofilms grown on different plates but from the same bacteria suspension). More details are given in Methods section.

The yield stress (τ_Y_) was also determined from the obtained amplitude sweeps as the product of the yield strain (γ_Y_) and the shear modulus *G* = √(*G*′^2^ + *G*″^2^) (Figure S3B). The yield stress represents the limit of the linear viscoelastic range, i.e., the amount of stress where irreversible plastic deformation begins to occur. Biofilms producing only curli displayed a lower yield stress than their counterparts where curli are associated with (pEtN-)­cellulose (Figure 2E). Interestingly, the biofilms containing both fibers present a yield stress below 43 Pa for AR3110 and 62 Pa for the mixture of W3110 and AP329. Biofilms lacking both fibers showed a yield stress of 60 Pa and biofilms producing only nonmodified cellulose resisted yielding up to 92 Pa, which is higher than any other ECM composition.

The flow stress (τ_F_) was derived as the flow strain (γ_F_) multiplied by the shear modulus *G* = √(*G*′^2^ + *G*″^2^) and corresponds to the stress where the viscous component begins to dominate over the elastic component (*G*′ < *G*″). At this point, more energy is irreversibly dissipated than reversibly stored and the material predominantly behaves like a fluid. Biofilms containing only curli or curli in association with nonmodified cellulose started to flow at 180 Pa. This was lower than homogenized biofilms with any other ECM composition, which began to flow at stresses larger than 400 Pa (Figure 2F).

### Influence of ECM Composition on the Local Mechanical Properties of Native Macrocolony Biofilms Measured by Microindentation

In contrast to oscillatory shear-rheology, which requires harvesting and homogenizing several biofilms for one bulk measurement, microindentation enables several local experiments on a single biofilm while preserving its native architecture. To assess the contribution of different ECM components and their architecture to the viscoelastic properties of the biofilms, we complemented our mechanical characterization with microindentation experiments in the center of intact biofilms. For each strain, biofilms for microindentation were grown in similar conditions as for the rheology experiments. Microindentation was performed with a 50 μm diamond indenter in the “air-indent” mode.^38^ In this mode, the measurement starts before reaching the biofilm so that the detection of the surface upon contact is facilitated after experiencing a slightly negative force (Figure 3A) (displacement δ = 0). Further tip penetration constitutes the loading phase. At the maximum indentation, a holding step of 10 s was set to measure the viscoelastic relaxation of the compressive stresses. Finally, the tip was retracted to the initial position far above the surface, while the corresponding adhesive forces were recorded during the detachment phase.

**Figure 3:**
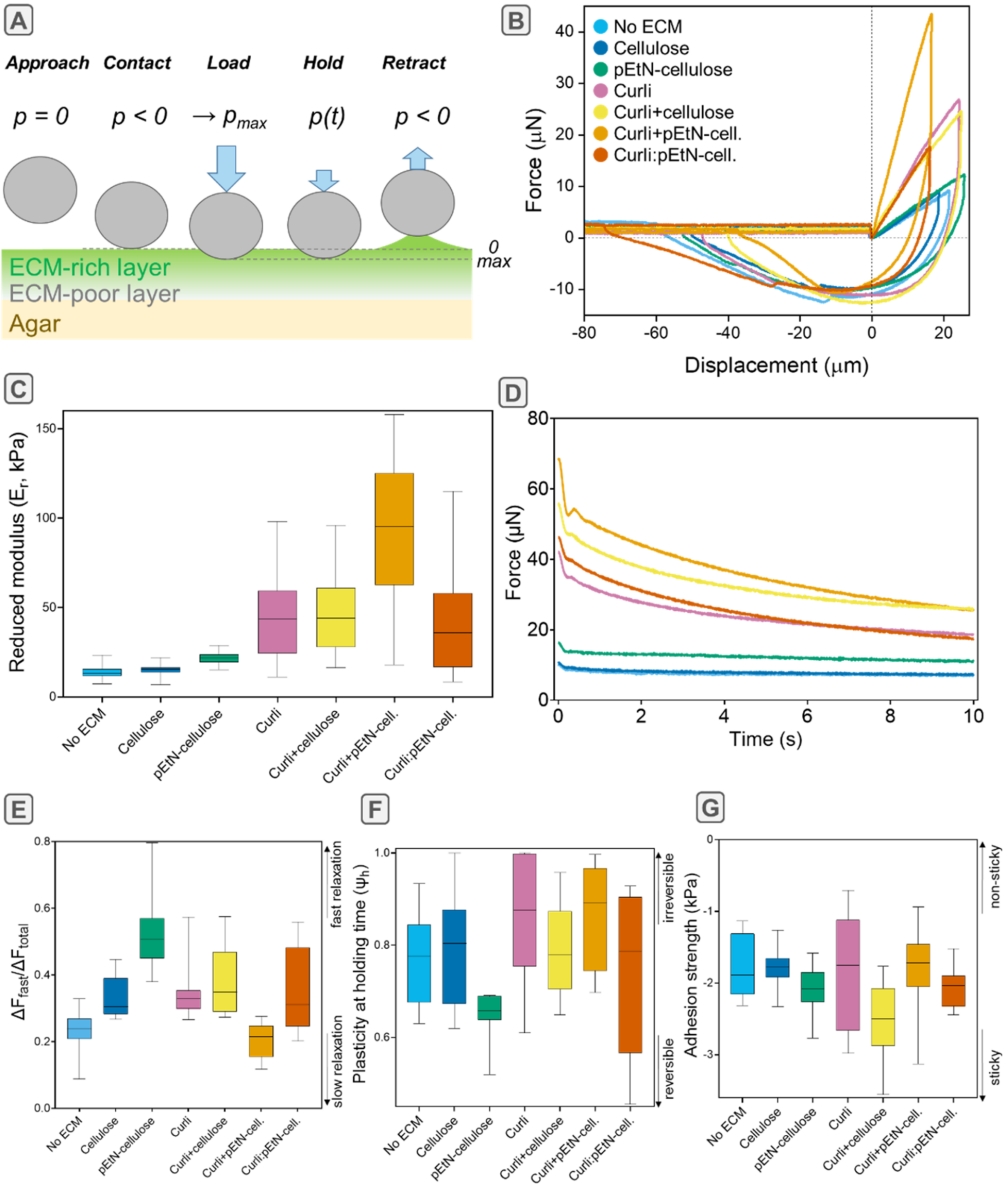
Mechanical characterization of macrocolony biofilms with microindentation. (A) Schematic image of the microindentation experiment performed on the native biofilms. (B) Representative force–displacement curves of biofilms with different ECM compositions, after the contact point is set at (0;0) for further analysis. (C) Reduced elastic moduli *E*_r_. (D) Representative force-time curves of each strain during the holding time at a maximum penetration depth of 20 μm. (E) Portion of the force relaxation happening before the tip instability Δ*F*_fast_/Δ*F*_total_, derived from the relaxation curves. (F) Plasticity during the holding time, defined as the ratio of energy dissipated during relaxation and energy stored upon indentation. (G) Adhesion strength σ_Adh_ measured from the minimum force recorded during tip retraction divided by the contact area at maximum indentation. Data in E–G are derived from indentation curves with a maximum penetration depth of 20 μm. See Methods section and Figure S3 for technical details. Median, quartiles and extreme values are represented by the thick line, the box limits and the whiskers respectively; *n* = 10 curves from *N* = 4 different biofilms. Statistical analysis was performed with a Mann–Whitney *U* test and is reported in Tables S5–8.

The slopes of the load–displacement curves characterize the stiffness of the biofilm material upon compressive loading (see Figure S4A and Methods section). Figure 3B shows that all biofilms producing curli reached higher maximum loads (*p*_max_) than curli-deficient strains when loaded to comparable indentation depths. The reduced elastic moduli (*E*_r_) were determined, fitting a Hertzian model for small displacements in the linear elastic region (<10 μm) (Figure 3C). The stiffest biofilms were those containing pEtN-cellulose and curli produced by the same bacteria (AR3110; 97 kPa), followed by the other biofilms containing curli (W3110, AP470, 1:1 mixture of W3110 and AP329) with values around 40 kPa. These values are about twice as high as those measured for all curli-deficient biofilms (Figure 3C). Statistical analyses suggested no difference between biofilms containing nonmodified cellulose or no ECM (Table S5). No significant differences were further observed between the mixed biofilm and biofilms obtained from the individual strains (W3110 or AP329) as well as between biofilms containing both nonmodified cellulose and curli produced by the same bacteria (AP470). These results indicate that the pEtN modification critically determines biofilm compressive stiffness.

As for many soft living materials, the mechanical properties of biofilms are expected to be time-dependent. To determine how their ECM composition and architecture affect their viscoelastic properties, relaxation was measured while holding the indenter for 10 s at the maximum displacement (Figure 3D). A local load maximum, consistently observed at 350 ms (Figure S4C), was attributed to a tip instability between 250 and 350 ms as the instrument switched from displacement-controlled indentation to constant displacement for the holding phase. Fast relaxation may be attributed to the flow of water in the porous network (<1 s) and slow relaxation to the reorganization of the ECM network itself (<10 s).^18^ We thus assessed the fast relaxation behavior by measuring the portion of force relaxed until the tip instability with respect to the total amount of force relaxed within the 10 s holding time (Δ*F*_fast_/Δ*F*_total_) (Figure 3D,E and Table S6). Most of the biofilms relaxed about 30 to 35% of the force before the tip instability. However, biofilms containing only pEtN-cellulose dissipated more than 50% of the total relaxed force in this short time window. In contrast, biofilms containing both curli and pEtN-cellulose formed by the same bacteria (AR3110) appeared to relax only 20% of the force in the short time window, thus suggesting that the relaxation is rather dominated by ECM rearrangement. Bacterial aggregates without ECM also showed a lower portion of force relaxation within the first 205 ms; here, most probably due to bacteria rearrangement. Note that such characteristic times remain short compared to biofilm growth and bacterial reorganization dynamics (>100 s),^39^ which are thus expected to play a minor role.

To compare the energy dissipated in different biofilms, we defined the plasticity during the holding time (Ψ_h_) as the ratio of the energy dissipated during relaxation and the total energy stored upon indentation (see Methods section). While all biofilms dissipated more than half of the energy stored (Ψ_h_ > 0.5), biofilms containing only pEtN-cellulose (AP329) consistently dissipated less compared to biofilms with other ECM compositions (Figure 3F and Table S7). Biofilms formed by the mixed strains were an exception but presented a broad distribution of plasticity values. In contrast, biofilms containing only curli (W3110) dissipated almost all energy during the holding time (Ψ_h_ = 0.93), which was similar to the energy dissipated by biofilms producing both curli and pEtN-cellulose formed by the same bacteria (AR3110) (Ψ_h_ = 0.85) but higher than for the other biofilms (see Table S7 for statistical significance values).

During the retraction phase, negative forces (*p* < −10 μN) and displacements (<−70 μm) were necessary to detach the tip from the biofilm surface. Values characterizing the adhesion strength were derived from the minimum forces measured during retraction divided by the maximum contact area between the indenter and the biofilm (i.e., at maximum displacement). While the broad distributions of adhesion strengths do not allow for clear conclusions, statistical analysis suggests that biofilms containing both curli and nonmodified cellulose were more adhesive than the others with an adhesion strength of 2.50 kPa (Figure 3G and Table S8). Moreover, biofilms with ECM made of pEtN-cellulose only appeared to adhere more than biofilms containing nonmodified cellulose only (2.08 kPa vs 1.79 kPa). This is another indication that pEtN-cellulose has an important contribution to the mechanical biofilm properties.

### Importance of Intact Macrocolony Biofilm Morphology, ECM Composition, and Fiber Arrangement

Microindentation showed a broad distribution of viscoelastic properties and the trends observed with this method did not always reflect those measured with bulk shear-rheology. To better understand these discrepancies, we first investigated the major structural differences between the various biofilms. We then focused on biofilms from producing curli and/or pEtN-cellulose and aggregates from producing no ECM to study how the respective biofilm architectures were affected by a homogenization step (mixing).

As previously shown, biofilm internal architecture highly depends on ECM composition.^36^ Here, fresh biofilms grown on salt-free LB agar, supplemented with Direct Red 23 (also known as Pontamine Fast Scarlet 4B), were sandwiched using a second layer of agar. Sections with a thickness of ∼1 mm were cut in the vertical direction along the biofilm diameter (± 1 mm precision), and the resulting cross sections were imaged with confocal microscopy (Figure 4). The obtained images provide a better understanding of differences in biofilm wrinkling and also reveal the internal organization of the ECM. As expected, samples from bacteria producing no ECM barely display a fluorescent signal. In agreement with the stereomicroscopy images (Figure 1A), biofilms containing only nonmodified cellulose were rather flat and biofilms containing both curli and pEtN-cellulose displayed many wrinkles with high aspect ratio (Figure 4). However, while biofilms containing only pEtN-cellulose appeared flat from above, the cross sections revealed a lot of small, thick and densely packed wrinkles, consistent with what has been previously described.^16^ This observation also agrees with their higher surface density (Figure S5 and Table S9). For biofilms containing only curli, very few wrinkles were seen both with stereomicroscopy and in the cross sections. Cross sections of biofilms containing curli and nonmodified cellulose displayed more wrinkles than biofilms containing only curli, but less than biofilms containing both curli and pEtN-cellulose. Further investigation of the microstructure of the stained ECM showed that the ECM appears granular in biofilms containing nonmodified cellulose only, curli only and the combination of both, while biofilms containing pEtN-modified cellulose showed a fibrous ECM with layers.

**Figure 4:**
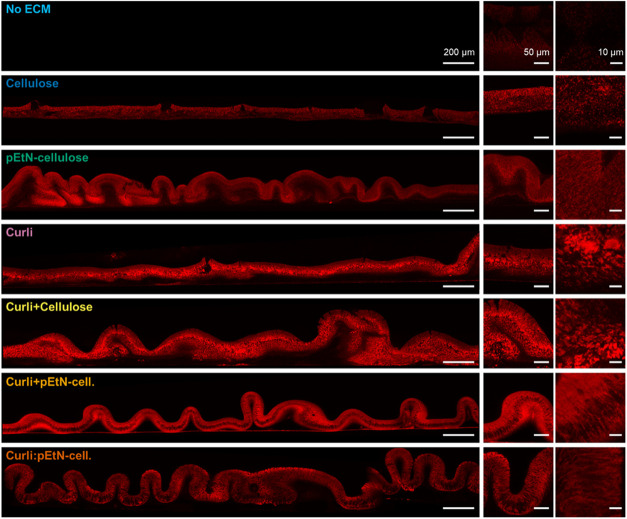
Structure of macrocolony biofilms grown from the different strains. Confocal images of cross sections of native biofilms grown at 28 °C for 7 days on salt-free LB agar supplemented with Direct Red 23 to stain the ECM (red). For each strain, the images are representative of images acquired for 3 biofilms seeded on different agar plates from the same bacterial suspension. The images were acquired with 552 nm laser excitation and photon collection in the range from 600 to 700 nm. All images are displayed with the same brightness and contrast settings.

One major difference between oscillatory shear-rheology and microindentation lies in the homogenization step required for bulk rheology. To assess the structural effect of biofilm scraping and mixing, the same imaging procedure was performed on homogenized biofilms, prepared as for the rheology experiment. Figure 5A presents a direct comparison of images taken for native vs homogenized biofilms, now focusing on bacteria that produce no ECM, only pEtN-cellulose, only curli or both of these ECM fibers. No structural change was visible when mixing biofilms containing none of the fibers. The densely packed patches observed in native biofilms containing curli only appeared to be more evenly distributed in the homogenized mass. The wrinkly morphology of biofilms containing pEtN-cellulose or pEtN-cellulose in combination with curli fibers was greatly affected by the mixing step. After mixing, the overall structure was destroyed and the folds were loosely pressed against each other. At the same time, the layer structure within the biofilms remained visible, suggesting partial homogenization of the macroscopic structure while the microscopic structure remained intact.

**Figure 5:**
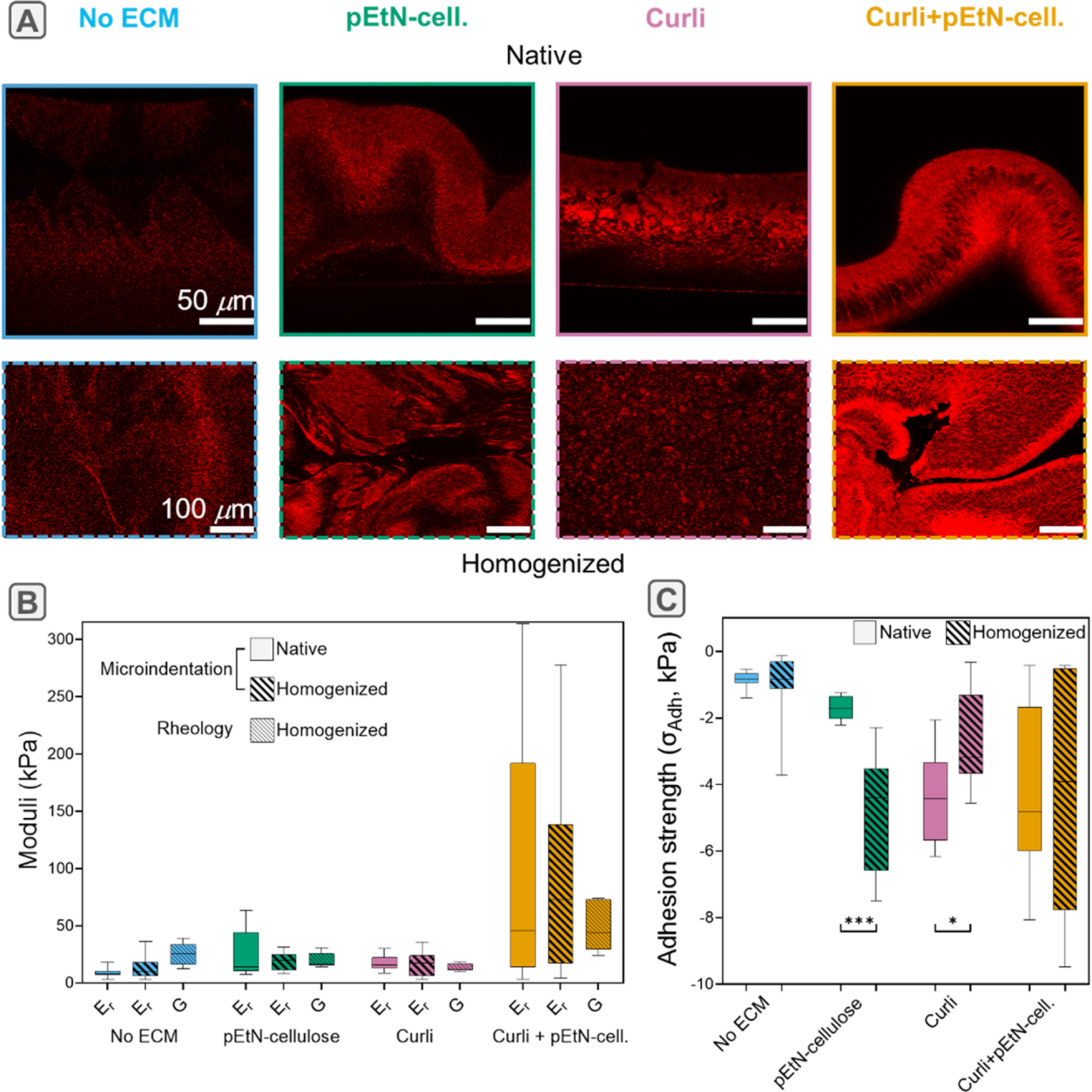
Influence of a homogenization step (mixing) on the structure and mechanics of selected macrocolony biofilms with different ECM composition. (A) Confocal images of native vs homogenized biofilms, formed by bacteria producing no ECM, only pEtN-cellulose, only curli or both ECM fibers. (B) Comparison of the reduced indentation elastic moduli *E*_r_ of native and homogenized biofilms with the shear modulus *G* obtained from rheology. Note that a quantitative comparison between *E*_r_ and *G* was not performed because of the unknown Poisson ratio. (C) Adhesion strength σ_Adh_ measured from the minimum load recorded during retraction. The values are normalized by the contact area at maximum indentation. Median, quartiles and extreme values are represented by the horizontal line, the box limits and the whiskers respectively; *n* = 7 curves from *N* = 3 different biofilms. Statistical analysis was performed using a Mann–Whitney *U* test and significance values are reported in Table S10.

To assess how structural changes upon homogenization affect biofilm mechanical characterization, we directly compared oscillatory shear-rheology and microindentation with biofilms grown on the same plates. This experimental design minimizes the contribution of plate-to-plate variability, frequently observed when growing biofilms. The compressive and shear moduli measured in this comparative experiment followed similar trends as those obtained with the two independent measurement campaigns described above. The median of the reduced indentation elastic moduli of native biofilms (i.e., conserving their original architecture) were the lowest for curli-deficient biofilms (∼7.9 to 13 kPa), slightly higher for curli-only biofilms (18 kPa) and significantly higher for biofilms containing both curli and pEtN-cellulose (26 kPa) (Figure 5B and Table S10). The rheology experiments showed a different trend. Homogenized samples containing no ECM or biofilm with both curli and pEtN-cellulose presented higher shear moduli (25 and 44 kPa, respectively) than biofilms with only pEtN-cellulose or curli (16.4 and 11.6 kPa, respectively).

To explore the role of sample preparation, this experiment also included microindentation on homogenized biofilms. Different mechanical parameters were compared between native and homogenized biofilms containing the different ECM compositions. No statistical difference was observed between the reduced moduli (Figure 5B and Table S10), the plasticity at holding time (Figure S6A and Table S10) nor the portion of force relaxation happening fast (Figure S6B and Table S10). However, a 2-fold increase in adhesion strength was observed for homogenized biofilms containing only pEtN-cellulose, as well as a slight decrease for biofilms containing only curli (Figure 5C and Table S10).

Figure 6 represents the shear moduli obtained from oscillatory shear-rheology (i.e., after sample homogenization) plotted against the reduced elastic moduli obtained with microindentation. Figure 6A shows data compiled from the shear-rheology and microindentation experiments reported in Figures 2 and 3. These experiments were performed independently, i.e., not at the same time and by different operators. Figure 6B shows data from comparative experiments where biofilms were grown on the same plate. Three plates were analyzed per selected strain, where biofilms were seeded from the same bacteria suspension to minimize biological variability. The dashed lines represent the theoretical relation between the shear modulus *G* and the reduced modulus $E_{r}=\frac{E}{\left(1-v^{2}\right)}=\frac{2G\left(1+v\right)}{\left(1-v^{2}\right)}$ for ideal materials with different Poisson ratios *v*.

**Figure 6:**
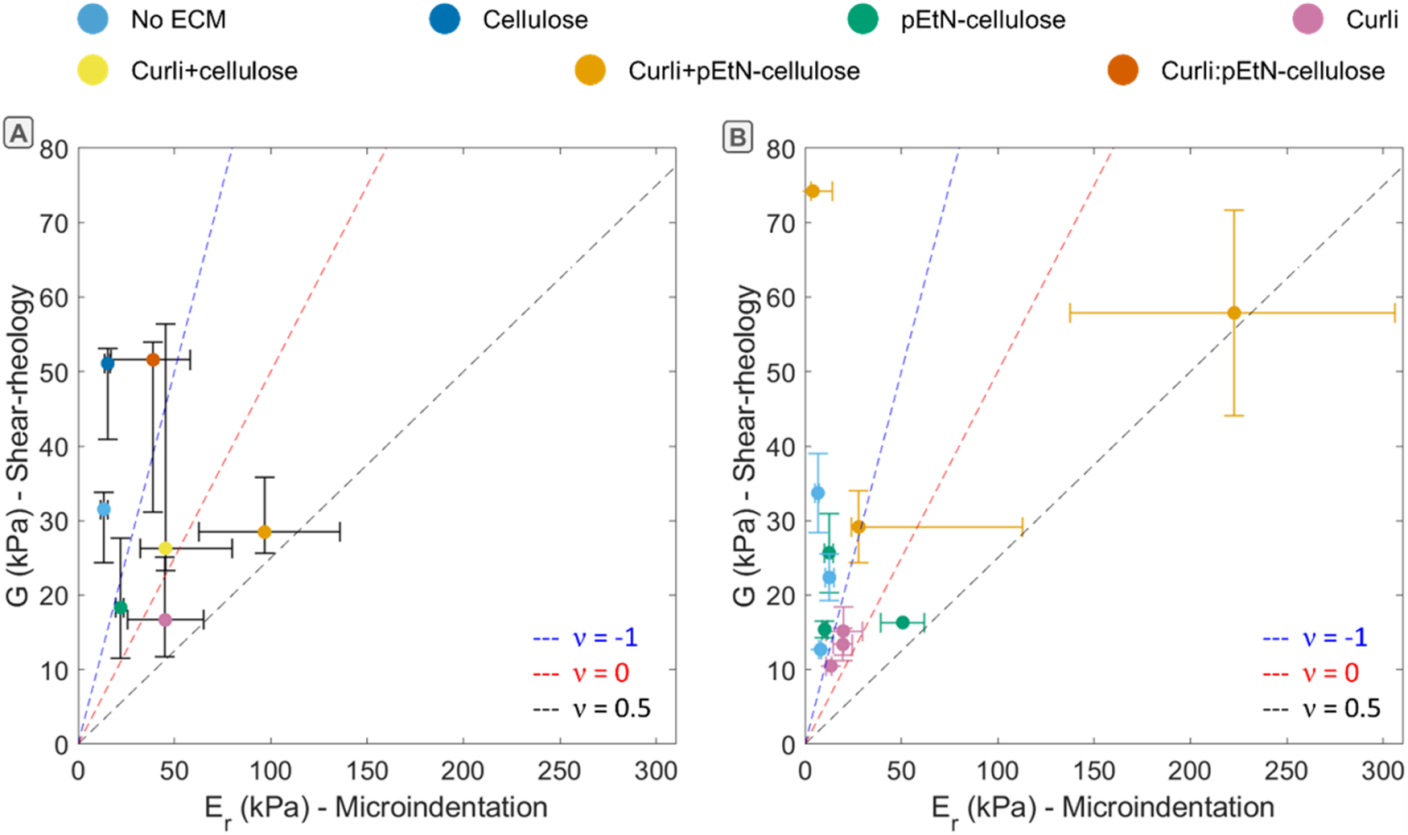
Shear modulus vs reduced elastic modulus obtained on macrocolony biofilms with different ECM compositions. Each point represents one median value. The error bars show the 0.25 and 0.75 quantiles of the data distribution. (A) The data are compiled from the independent experiments reported in Figures 2 and 3. (B) The data correspond to the comparative experiments performed with biofilms grown on the same plate (*N* = 3 plates, *n* = 3 biofilms per plate). The dashed lines represent theoretical relations between the shear modulus *G* and the reduced modulus *E*_r_ for ideal materials with different Poisson ratios.

The scattered positions of the experimental points with respect to these lines confirm that biofilms are complex materials with nontrivial mechanical behavior. This result also suggests that the experiments become hardly comparable to the theory after modifying biofilm internal structure through homogenization. Another observation is the wide distribution of moduli, both for the rheology and the microindentation data, despite limiting plate-to-plate variations. This observation was especially striking for data obtained for biofilms grown from bacteria producing both curli and pEtN-cellulose. In contrast, biofilms deficient in curli seem to yield narrower distributions of reduced moduli obtained by microindentation. Aware of possible variations in agar properties from plate to plate, we verified that there was no systematic effect of the agar reduced modulus on biofilm reduced modulus (Figure S7). Despite the large distribution of values, biofilms containing none or only one type of fiber tend to have low moduli (the corresponding points are located at the bottom left of Figure 6), whereas biofilms containing curli and (modified-)­cellulose tend to have larger moduli (top right).

## Discussion

This work reports on the mechanical analysis of macrocolony biofilms formed by a collection of strains that produce ECM of different compositions. Comparing oscillatory shear-rheology and microindentation, we found that microindentation yields more conclusive results. Indeed, microindentation, which conserves the native structure of the biofilms, indicates that biofilms containing curli fibers tend to be stiffer and that the pEtN-modification of cellulose enhances biofilm stiffness further when curli fibers are present.

Oscillatory shear-rheology and microindentation are two distinct mechanical characterization methods that fundamentally differ in two key aspects. Rheology applies a shear stress to the sample, whereas microindentation predominantly probes it under compressive stress. Additionally, bulk rheology averages the properties of one entire or several biofilms, while microindentation locally probes the biofilm over a region that encompasses numerous bacteria and their surrounding ECM (∼3000 to 7000 μm^2^). A quantitative comparison of the shear and compressive moduli requires knowledge of the Poisson ratio, which is not easily available for such highly heterogeneous materials. However, biofilms are not necessarily expected to exhibit similar mechanical responses under these different conditions and at the scales analyzed. This highlights the complementarity of the two methods, especially for investigating the differential mechanical responses of biofilms grown from different strains, as demonstrated in this work.

The sample preparation strategies chosen are also very different for the two methods. It is in principle possible to transfer intact macrocolony biofilms from the agar plate directly to the rheometer; however, the heterogeneity of the sample is rarely taken into account and biofilm wrinkles are compressed while reaching the final gap size.^40^ In the literature, homogenization steps are scarcely applied to reduce potential variability due to a random positioning of the biofilm structure when scrapping and transferring the sample from the agar to the rheometer.^41^ Here, we gently mixed the samples to obtain a homogenized and compact biofilm mass between the rheometer plates.^23^ As a consequence, the measured viscoelastic properties represent a biofilm material where the macroscopic structure was largely destroyed. As the structure was locally retained in small volumes (Figure 5A), it is unclear which interactions determine the average viscoelastic properties of the homogenized biofilm material. In contrast, the original morphology and internal structure are conserved when measuring macrocolony biofilms with microindentation. While this approach accounts for the contribution of biofilm architecture to the mechanical properties measured, the many wrinkles present on biofilms containing pEtN-cellulose, especially when combined with curli, may contribute to the large distribution of values for the different mechanical parameters, e.g., due to local buckling of wrinkles upon indentation.^16^ To reduce variations caused by the three-dimensional morphology, we indented biofilms in their central region, where wrinkles are less pronounced and less likely to affect the indentation curves. To mitigate the possible influence of biofilm morphology, we also increased the number of statistical repeats to 10 measurements per biofilm. The positions of these measurements were separated by at least 250 μm to avoid any cross-influence between measurement points. The experiment comparing microindentation on native vs homogenized samples (Figure 5B) shows no significant differences for all the strains tested, irrespective if they show wrinkles (e.g., pEtN-cellulose and Curli + pEtN-cell.) or not (e.g., No ECM and Curli). This observation supports that the local reduced elastic moduli obtained in flat areas are representative of the biofilm mechanical properties.

These technical difficulties related to the complexity of the biofilm material and its morphology add to the variability inherent to biofilm research that has been reported for mechanical studies.^35^ Such variability can be the consequence of subtle changes in experimental conditions, e.g., the atmospheric conditions in the laboratory that can, in turn, affect the (micro)­conditions of growth for the bacteria in the agar plate. Indeed, despite following the same protocols as in the literature, the differences in biofilm morphologies, obtained for the different strains, were not as marked as those reported.^36,42^ Moreover, we noticed plate-to-plate variations that we could not attribute to variations in the agar mechanical properties (Figure S7). Yet, we accounted for possible influences by growing the different strains in one and the same plate, except for the experiment presented in Figure 5, where several macrocolony biofilms from one strain were grown in the same plate to compare the two characterization methods. Considering the wide distribution of results, the trends observed in the independent experiments where microindentation and shear-rheology were performed on biofilms from different plates (Figure 6A), were notably coherent and we are thus confident that the observed differences in mechanical parameters reflect the properties of the biofilms. While no significant influence of homogenization was measured on the reduced indentation elastic moduli of biofilms from different strains (Figure 6B), we found slightly different trends between strains when comparing the shear moduli obtained by shear-rheology (Figures 2C,D and[Fig fig5]B) and the reduced elastic moduli obtained by microindentation (Figures 3C and[Fig fig4]B). This observation could indicate a major interplay between the loading geometry and biofilm structure during the mechanical test, especially for the samples with no ECM. Differential changes of biofilm adhesion strength observed upon homogenization, as a function of ECM composition (Figure 5D) may also explain the differences observed while comparing microindentation and shear-rheology. Indeed, while adhesion is not expected to impact the response of the material to compression, it may contribute to the response of the material to shear forces.

Mechanical data obtained at subcellular scales may help to understand biofilm mechanical behavior as a function of their composition. For example, Kreis et al.^30^ performed AFM nanoindentation on AR3110 biofilms and identified two populations of ECM with distinct elastic properties (Young’s moduli of ∼1 and 10 kPa, respectively). Taken together with previous observations reported in,^37^ the present results obtained on biofilms from mutants producing either curli or pEtN-cellulose or both types of fibers suggest that the stiffer ECM of AR3110 corresponds to curli fibers while the softer ECM corresponds to pEtN-cellulose. Interestingly, bacteria measured at the subcellular scale are very stiff (∼1 to 10 MPa),^30^ although the biofilms grown from deprived of ECM appeared to be the softest of our collection with ∼10 kPa (Figure 3C). In contrast to nanoindentation with AFM, which measures individual bacteria, microindentation on biofilms rather probes their mechanical interactions and how these are mediated by the presence of ECM. As such, a collection of stiff bacteria that are not decorated with ECM fibers is expected to locally rearrange and dissipate energy under minimal compressive load (Figure 3C,D), while bacteria embedded in curli and/or pEtN-modified cellulose may further transmit forces over larger distances within the biofilm and partially store energy in their ECM. In both cases, the relatively small proportion of force dissipated during the first 205 ms of the holding time indicates that energy dissipation rather occurs via bacteria and/or ECM rearrangement than via water flow (Figure 3E).

Our results also suggest that the ability of bacteria to retain or rearrange their organization under mechanical stress depends on the physicochemical properties of their surrounding ECM. For example, cellulose fibers only appear to provide a mechanical advantage to the colony if it is modified with the zwitterionic phosphoethanolamine (pEtN) group. The pEtN-modification not only slightly increases biofilm elastic modulus (Figure 3C) but also significantly speeds up force relaxation (Figure 3E) and slightly increases adhesion (Figure 3F) compared to nonmodified cellulose. On the other hand, the presence of curli alone notably increases biofilm rigidity at the expense of a higher plasticity, indicating that the material better resists mechanical stress while it is more prone to irreversible deformation. Finally, our results indicate that biofilms containing both curli and pEtN-cellulose are only stiffer than those containing curli only when the fibers are assembled by the same bacteria (orange data points, Figure 3C). This clear increase of rigidity suggests that the biofilm matrix is a composite material. It is not observed when curli are combined with nonmodified cellulose. Our study thus quantitatively confirms that the natural pEtN-modification has significant mechanical implications at the biofilm level in wild-type strains (AR3110), which is consistent with the observed morphology of macrocolony biofilms (Figures 1A and 4).^36^ Indeed, wrinkling theory predicts more surface instabilities in stiffer (bio)­films, given a similar strain mismatch and adhesion energy at the interface.^43^ The mechanical analysis of biofilms with different combinations of curli and pEtN-cellulose or nonmodified cellulose also highlights the importance of when and how the different ECM fibers interact with each other or maybe even coassemble. When comparing biofilms where curli and pEtN-cellulose are produced by the same bacteria or by different subpopulations, a lower stiffness is observed when the fibers are produced by different bacteria (Figure 3C). While the two strains used to form mixed biofilms have similar growth rates due to their nearly identical genetics (differing only in their specific matrix components), bacteria producing only one type of fiber may phase separate during biofilm growth, resulting in regions with distinct properties.^44^ Despite recent work performed with synthetic pEtN-cellulose, the finely tuned interaction between curli and pEtN-cellulose remains poorly understood at the molecular level and is the subject of ongoing work.^45,46^

Lastly, our data suggest that the association of curli and pEtN-cellulose enables to build a tunable composite material that can span a wide range of mechanical properties, potentially using only little variations in composition (i.e., curli to pEtN-cellulose ratio) (Figure 4). This is consistent with the existence of intracellular levels of regulation, affecting either one or both fibers,^47^ as well as extrinsic compounds targeting specifically one fiber.^48^ While the large spread in our data could be a consequence of the heterogeneity and morphology of the biofilms as discussed earlier,^15,30^ it was also visible in the microindentation experiments performed on homogenized biofilms (Figure 5B) and in rheology experiments (Figure 6). The presence of both curli and pEtN-cellulose as building blocks may thus allow to finely adjust the biofilm properties in response to the environment.

Overall, our mechanical comparison of macrocolony biofilms with altered ECM composition quantitatively confirms the key role of curli fibers in biofilm rigidity. It also highlights the crucial contribution of pEtN-cellulose, and especially of the pEtN modification, to the mechanical properties and structural stability of biofilms, especially when associated with curli fibers. While further work at the molecular and fiber levels is needed to understand the interactions between biofilm ECM components, our study provides useful insights into how these interactions impact macrocolony biofilm mechanics. The knowledge presented here is of particular interest for researchers aiming at bridging the gap between ECM production, assembly and hierarchical structure up to macroscopic biofilm materials properties and functions, be it for preventing biofilm formation or engineering biofilms for technical purposes.^49,50^

## Methods

### Bacterial Strains and Growth

All strains are derived from the strain W3110, which synthesizes amyloid curli protein but no pEtN-cellulose (Table S1).^16^ Curli amyloid fibers are macromolecular assemblies of CsgA (as the major subunit), and CsgB (as a minor subunit). The strain AR3110 is a derivative of W3110 with a restored capacity to produce phosphoethanolamine (pEtN)-modified cellulose.^16^ Thus, AR3110 is a highly proficient biofilm-forming strain that produces both amyloid curli protein and pEtN-cellulose as major ECM components (curli + pEtN-cellulose). AP329 (*csgBA*::kan) is an AR3110 derivative that is deficient in the production of curli while producing pEtN-cellulose.^16^ This strain has a kanamycin resistance cassette associated with the mutation in the structural curli operon (*csgBA*). AP472 (*bcsG*::scar, *csgBA*::kan) is an AR3110 derivative that is deficient in the production of curli and that produces nonmodified cellulose (i.e., without the pEtN-modification).^16^ This strain also has a kanamycin resistance cassette associated with the mutation in the structural curli operon (*csgBA*). AP470 (*bcsG*::scar) is an AR3110 derivative that produces amyloid curli protein and nonmodified cellulose.^16^ AR198 (*bcsA*::scar, *csgB*::cm) is also an AR3110 derivative that is deficient in the production of both curli and pEtN-cellulose.^16^ This strain has a chloramphenicol resistance cassette associated with the mutation in the curli structural gene *csgB*. In this work, we also refer to AR198 as “No ECM” (Figure 1A). It may still produce ECM components other than curli and pEtN-cellulose, which are usually less abundant (e.g., pga and colanic acid).^51,52^ For the samples termed “co-seeded” (curli:pEtN-cellulose), the strains W3110 and AP329 were mixed in a 1:1 ratio upon seeding. As previously described, OD_600_ was measured for each liquid culture containing one of the strains.^44^ In the case that their OD_600_ was not identical, both cultures were mixed such that the cell density of each strain was identical in the suspension used for seeding. Inoculation on salt-free agar took place immediately after mixing.

Petri dishes (15 cm diameter) were filled with 100 mL of salt-free agar, composed of 1.8 w/v% bacteriological grade agar–agar (Roth, #2266), 1 w/v% tryptone (Roth, #8952) and 0.5 w/v% yeast extract (Roth, #2363). The agar plates were kept in ambient conditions for 48 h before use. Bacteria were initially streaked out on LB agar (Luria/Miller, Roth, #969) and grown overnight at 37 °C. Liquid cultures were subsequently started from a single colony, using liquid LB medium (Roth, #968). Bacteria were again grown overnight at 37 °C with shaking at 250 rpm. Finally, each plate was inoculated with arrays of four or nine drops (5 μL) of bacterial suspension (OD_600_ ∼ 0.5 after 10× dilution). After inoculation, the excess of water evaporated from the drops and bacteria-rich areas of comparable size (diameter of approximately 3 to 4 mm) were visible on the surface. The biofilms were finally grown for 7 days at 28 °C.

### Biofilm Area, Dry Mass, Density, and Water Content

After 7 days of growth, 6 out of 9 biofilms per strain were randomly selected and imaged in bright field with a stereomicroscope (AxioZoomV.16, Zeiss, Germany) using the tiling function of the acquisition software (Zen 2.6 Blue edition, Zeiss, Germany). Biofilm size was quantified by calculating their projected area using a custom-made MATLAB code based on thresholding.^31^ The water content of the biofilms was determined by scraping 9 biofilms per condition from the respective agar substrates. Biofilms were placed in plastic weighing boats, and dried in an oven at 60 °C for 3 h. Wet and dry masses (m_wet_, m_dry_) were determined before and after drying and used to calculate the water content as W = (m_wet_ – m_dry_)/m_dry_ × 100%w/w.^53^ To calculate biofilm density, the previously calculated areas were used. Assuming the biofilms have a consistent thickness and distribution across the plate, the mean dry mass of the plate (divided by 9 to approximate the unit mass of each biofilm) was divided by the calculated area of each biofilm to obtain the density, expressed in mg.cm^–2^. All procedures were carried out in four independent experiments.

### Oscillatory Shear-Rheology of Biofilms

Biofilms were either measured directly after growth or stored in the fridge for less than 48 h. For storage at 4 °C, the Petri dishes were sealed with parafilm to prevent evaporation. Depending on the strain, two or three biofilms, corresponding to a mass of ∼90 mg, were harvested from the agar surface using cell scrapers, transferred into a Petri dish and homogenized by stirring for 30 s. Three measurements were acquired per strain, all with biofilms grown on different plates, but from the same bacteria suspension.

The rheometer used was a stress-controlled shear rheometer (MCR301, Anton Paar GmbH, Ostfildern, Germany). The bottom plate was equipped with Peltier thermoelectric cooling and the temperature was held constant at 21 °C for every experiment. Once the sample was transferred onto the bottom plate, a channel around the plate was filled with water. A hood was lowered down onto the plate to obtain a closed measurement chamber and maintain a high humidity level. A parallel plate geometry with a top plate of 12 mm diameter was used for every experiment. The gap height between the plates was set to 250 μm. Using oscillatory shear measurements, an amplitude sweep was performed to determine the linear viscoelastic range (LVE) and to extract the plateau values of the storage (*G′*_0_) and loss (*G″*_0_) moduli. The oscillation frequency was 10 rad s^–1^. The strain amplitude was first increased stepwise from 0.01 to 100% with seven points per decade and then decreased again to 0.01%. These cycles were repeated three times per experiment (i.e., six intervals). One complete experiment lasted approximately for 45 min. The data presented in the Results section were extracted from the second interval with increasing strain amplitude. The first cycle was considered as one additional homogenization step of the biofilm. The plateau values *G′*_0_ and *G″*_0_ represent the mean values over a strain range from 0.01 to 0.02%, corresponding to three measurement points. We verified that storing the samples at 4 °C for 48 h did not cause any systematic increase or decrease in the measured moduli.

### Microindentation on Biofilms

Freshly grown biofilms were used for the indentation experiments. For each strain (incl. the coseeded W3110 and AP329 strains), ten measurements were performed in the central region of four biofilms each. The distance between two measurement points was at least 250 μm in *x* and *y* directions. The indentation depth was between 10 and 30 μm, i.e., much less than the biofilm thickness (∼100 μm).^54^ A TI-950 Triboindenter (Bruker Hysitron, Eden Prairie, United States) equipped with an extended stage (XZ-500) and a cono-spherical tip (*R* = 50 μm) were used to determine the load–displacement curves using the “air-indent” method.^38^ Loading rates were set at 20 μm s^–1^, which translates into loading and unloading times of 10 s. The loading portion of all curves were fitted with a Hertzian contact model over an indentation range of 0 to 10 μm to obtain the reduced elastic modulus *E*_r_ (eq 1).^31^

1 $$p=\frac{4}{3}E_{r}R^{1/2}\delta^{3/2}$$

where *p* is the contact force in μN, *E*_r_ is the indentation elastic modulus, *R* is the tip radius, and δ is the contact depth. To characterize biofilm relaxation behavior, load-time curves were extracted during the holding period. A local load maximum was consistently observed at 350 ms. It can be attributed to a tip instability, occurring between 250 and 350 ms, when the instrument switches from displacement-controlled indentation to constant displacement for the holding phase. From this curve, we calculated the portion of force relaxation happening before the tip instability (eq 2)­

2 $$\frac{\Delta F_{fast}}{\Delta F_{total}}$$

where Δ*F*_fast_ represents the fast relaxation reaction occurring between *t* = 0 and 205 ms (Figure S4C). Δ*F*_fast_ was quantified following eq 3.

3 $$\Delta F_{\mathrm{fast}}=F_{\max}-F_{205\mathrm{ms}}$$

We define *F*_Total_ as the total force relaxed after 10 s (end point for the holding time), following eq 4.

4 $$\Delta F_{total}=F_{\max}-F_{relaxed}$$

The plasticity index at the holding point (ψ_h_) is defined in eq 5 (Figure S4B)­

5 $$\psi_{h}=\frac{A_{1}}{A_{total}}$$

where *A*_1_ describes the area between the loading and unloading curves during the holding period, and *A*_total_ describes the area under the loading curve. ψ_h_ spans from 0 to 1, where ψ_h_ = 1 means that the biofilm is fully plastic, and ψ_h_ = 0 means that the biofilm is elastic.

For each biofilm, a subset of curves with an indentation depth between 10 and 25 μm were selected for the analysis of the adhesion force and viscoelastic stress relaxation.

The adhesion strength (σ_Adh_) is defined in eq 6 (Figure S4)­

6 $$\sigma_{\mathrm{Adh}}=\frac{F_{\min}}{A_{\mathrm{contact}}}$$

where *F*_min_ is the force applied in the detachment point when the tip separates from the biofilm, and *A*_contact_ is the contact area between the tip and the biofilm at the largest indentation *h*_max_ reached (Figure S4D). *A*_contact_ is defined by eq 7.

7 $$A_{\mathrm{contact}}=2\pi Rh_{\max}$$

For the analysis of the relaxation curves, plasticity and adhesion strength (Figure 3) only indentation curves that did not exceed 20 μm penetration depth were considered. This was done to avoid any influence of the underlying agar as well as possible deviations from geometrical assumptions on the contact area between the indentation tip and the biofilm.

### Imaging of Biofilm Cross Sections

Biofilms were grown on agar plates supplemented with the fluorescent dye Direct Red 23 (Pontamine Fast Scarlett 4B) (CAS 3441–14–13, Sigma-Aldrich, Germany), using a final concentration of 0.03 g L^–1^.^55^ Cross sections of living biofilms were obtained as described previously by Siri et al.^53^ Briefly, liquid plain agar (1.8% w/v) at about 42 °C was slowly poured over each biofilm. The resulting agar–biofilm–agar sandwiches were cut into ∼1 mm-thick slices using a blade. The cross sections were placed onto glass coverslips. The different cross sections were observed with a confocal microscope (SP8 FALCON, Leica, Mannheim, Germany), using a 63× oil immersion objective (1.2 NA). Direct Red 23 fluorescence was excited at 552 nm and the emitted photons were collected in the range from 600 to 700 nm. Images were taken and analyzed with the LAS X software (Leica). Four cross sections were imaged per biofilm (3 independent biofilms from different agar plates, grown from one microcolony).

To image cross sections of homogenized biofilms, the biofilms were first treated as described in the rheology section. The homogenized biofilms were then transferred into 2 mm wells punctured into a Petri dish of plain agar gel (1.8% w/v) and covered with another layer of agar. The resulting agar–homogenized biofilm–agar sandwiches were cut into ∼1 mm-thick slices using a blade and imaged with a confocal microscope.

A wrinkling coefficient was determined from the cross sections and defined as (*L*_w_ – *L*_0_)/*L*_0_, where *L*_w_ is the actual length of the path following the wrinkled biofilm and *L*_0_ is the length of the straight line spanning the extremities of this path (Figure S5).

### Statistical Analysis

To analyze the different parameters obtained from shear-rheology, i.e., plateaus of the storage (*G*′_0_), loss (*G*″_0_), and shear moduli (*G*), yield stress (τ_Y_) and flow stress (τ_F_), the respective median errors were calculated by bootstrapping. From each original data sample X of size 3 (i.e., 3 independent measurements per bacteria strain), 3 elements were chosen with replacement to create a bootstrapped sample Y of size 3. The median *m* of Y was calculated and stored in the vector M of size 27. The previous steps were repeated for all 27 (= 33) combinations, hence the size of M. The standard deviation of M was taken as the error associated with the median of X.

For each microindentation experiment, *N* = 3 or 4 biofilms were tested and *n* = 7 or 10 curves were acquired per biofilm (see figure captions). The mechanical data was plotted and analyzed using the GraphPad Prism 9.3.1 software. Mann–Whitney *U* tests were used for statistical analysis (*p* < 0.0001, **** | *p* < 0.001, *** | *p* < 0.01, ** | *p* < 0.05, * | ns = nonsignificant).

## Supplementary Material



## Notes

Statistical significances of biofilm area, dry mass, surface density and mechanical properties; biofilm water content and water activities; shear modulus *G*_0_ calculated from *G*′_0_ and *G*″_0_; microindentation curves; quantification of biofilm wrinkling; influence of a homogenization step on biofilm mechanical properties; comparative plot of reduced elastic moduli of biofilms vs agar plates (PDF)
