# Supplementary material for: Mechanical Comparison of Biofilms with Altered Matrix Composition: A Study Combining Shear-Rheology and Microindentation
Source: ACS Biomater Sci Eng. 2025 Jun 12;11(7):4523–36. doi: 10.1021/acsbiomaterials.5c00261 (PMC12264770; doi:10.1021/acsbiomaterials.5c00261)
Supplement: Supplementary file 1 [file ab5c00261_si_001.pdf]

Mechanical comparison of *Escherichia coli* biofilms with altered matrix composition: a study combining shear-rheology and microindentation

Macarena Siri[1], Adrien Sarlet[1*], Ricardo Ziege[1*], Laura Zorzetto[1], Carolina Sotelo Guzman[1], Shahrouz Amini[1], Regine Hengge[2], Kerstin G. Blank[1,3#], Cécile M. Bidan[1#]

[1] *Max Planck Institute of Colloids and Interfaces, 14476 Potsdam, Germany*

[2] *Humboldt University, 10115 Berlin, Germany*

[3] *Johannes Kepler University Linz, Institute of Experimental Physics, Altenberger Str. 69, 4040 Linz, Austria*

* *Equal contributions*

[#] *Corresponding authors*: cecile.bidan@mpikg.mpg.de ; kerstin.blank@jku.at

Supplementary Information

E. coli strains used in this study	Table S1
Biofilm area: statistical significance	Table S2
Biofilm dry mass: statistical significance	Table S3
Biofilm density: statistical significance	Table S4
Biofilm water contents and water activities	Figure S1
Shear-rheology: amplitude sweeps of 3 individual experiments	Figure S2
tan δ_0 and shear moduli G_0 calculated from G'_0 and G''_0	Figure S3
Microindentation curves	Figure S4
Biofilm mechanical properties: statistical differences	Tables S5-S8
Quantification of biofilm wrinkling	Figure S5
Wrinkling coefficient: statistical significance	Table S9
Influence of a homogenization step on biofilm mechanical properties	Figure S6
Native vs. homogenized biofilms: statistical significance	Table S10
Comparative plot of reduced elastic moduli of biofilms vs. agar plates	Figure S7

Table S1. *E. coli* strains used in this study.

Strain Name	Extracellular Matrix	Comment	Reference
E. coli K-12 W3110	**curli**, no cellulose	due to a stop codon in *bcsQ*	Hayashi, 2006,[1] Serra, 2013.[2]
E. coli K-12 AR3110	**curli, pEtN-cellulose**	stop codon in bcsQ was replaced by a sense codon	Serra, 2013.[2]
E. coli K-12 AP329	no curli, **pEtN-cellulose**	AR3110 *csgBA*::*kan*	Thongsomboon, 2018.[3]
E. coli K-12 AP472	no curli, **non modified cellulose**	AR3110 *bcsG*::*scar*, *csgBA*::*kan*	Thongsomboon, 2018.[3]
E. coli K-12 AP470	**curli, non modified cellulose**	*AR3110 bcsG*::*scar*	Thongsomboon, 2018.[3]
E. coli K-12 AR198	no curli, no cellulose	*AR3110 bcsA*::*scar*, *csgB*::*cm*	Thongsomboon, 2018.[3]

1. Hayashi K, Morooka N, Yamamoto Y, et al. Highly accurate genome sequences of *Escherichia coli* K-12 strains MG1655 and W3110. *Mol Syst Biol*. 2006;2. doi:10.1038/MSB4100049
2. Serra DO, Richter AM, Hengge R. Cellulose as an architectural element in spatially structured *Escherichia coli* biofilms. *J Bacteriol*. 2013;195(24):5540-5554. doi:10.1128/JB.00946-13
3. Thongsomboon W, Serra DO, Possling A, Hadjineophytou C, Hengge R, Cegelski L. Phosphoethanolamine cellulose: A naturally produced chemically modified cellulose. *Science (80)*. 2018;359(6373):334-338. doi:10.1126/science.aao4096

Table S2. Statistical significance for Fig. 1B; Biofilm area.

Water Content (%w/w)	No ECM	Cellulose	pEtN-cellulose	Curli	Curli +cellulose	Curli +pEtN-cell.	Curli :pEtN-cell.
No ECM		ns	ns	ns	ns	ns	ns
Cellulose	ns		ns	ns	ns	ns	ns
pEtN-cellulose	ns	ns		ns	ns	ns	ns
Curli	ns	ns	ns		ns	ns	ns
Curli+cellulose	ns	ns	ns	ns		ns	ns
Curli+pEtN-cell.	ns	ns	ns	ns	ns		ns
Curli:pEtN-cell.	ns	ns	ns	ns	ns	ns	

Table S3. Statistical significance for Fig. 1C; Biofilm dry mass.

Dry mass (mg)	No ECM	Cellulose	pEtN-cellulose	Curli	Curli +cellulose	Curli +pEtN-cell.	Curli :pEtN-cell.
No ECM		ns	ns	ns	ns	ns	ns
Cellulose	ns		ns	ns	ns	ns	ns
pEtN-cellulose	ns	ns		ns	ns	ns	ns
Curli	ns	ns	ns		ns	ns	ns
Curli+cellulose	ns	ns	ns	ns		ns	ns
Curli+pEtN-cell.	ns	ns	ns	ns	ns		ns
Curli:pEtN-cell.	ns	ns	ns	ns	ns	ns	

Table S4. Statistical significance for Fig. 1D; Biofilm density.

Density (mg/cm^{-2})	No ECM	Cellulose	pEtN-cellulose	Curli	Curli +cellulose	Curli +pEtN-cell.	Curli :pEtN-cell.
No ECM		ns	***	*	*	ns	ns
Cellulose	ns		**	ns	*	ns	ns
pEtN-cellulose	***	**		****	****	**	***
Curli	*	ns	****		ns	ns	ns
Curli+cellulose	*	*	****	ns		ns	*
Curli+pEtN-cell.	ns	ns	**	ns	ns		ns
Curli:pEtN-cell.	ns	ns	***	ns	*	ns	

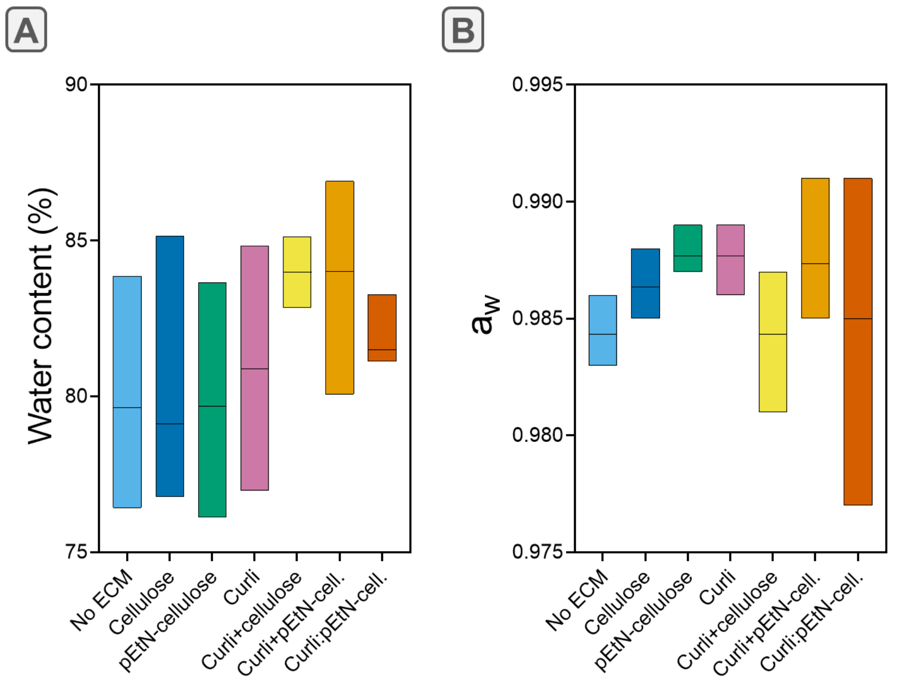

Figure S1. Biofilm water contents from N=4 plates per strain (A) and water activities from n=3 independent biofilms per strain, grown on different plates from the same microcolony (B). Differences were not statistically significant.

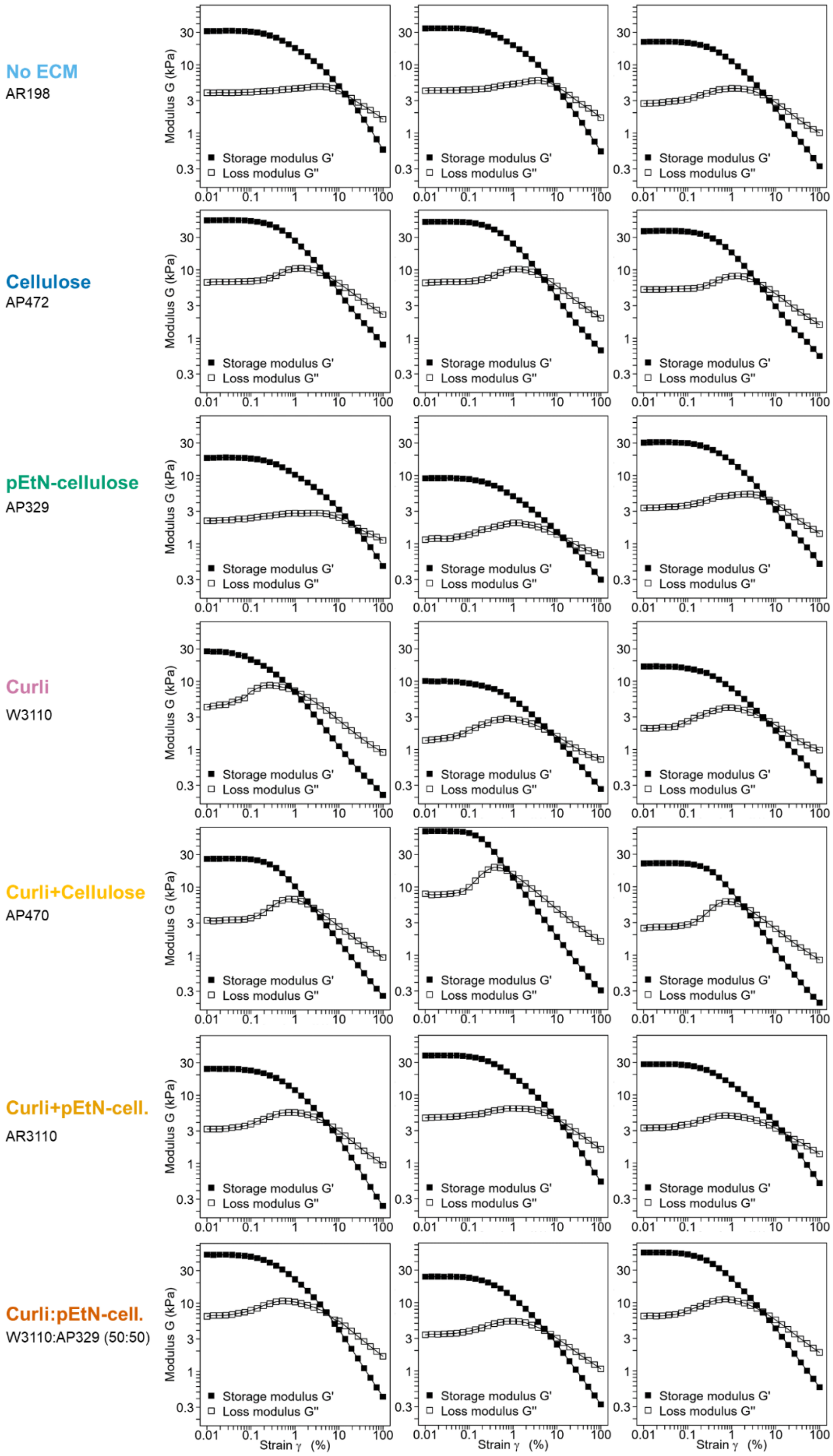

Figure S2. Amplitude sweeps of all different strains and mixtures (3 samples per condition), measured at an angular frequency of $\omega = 10$ rad s^{-1}.

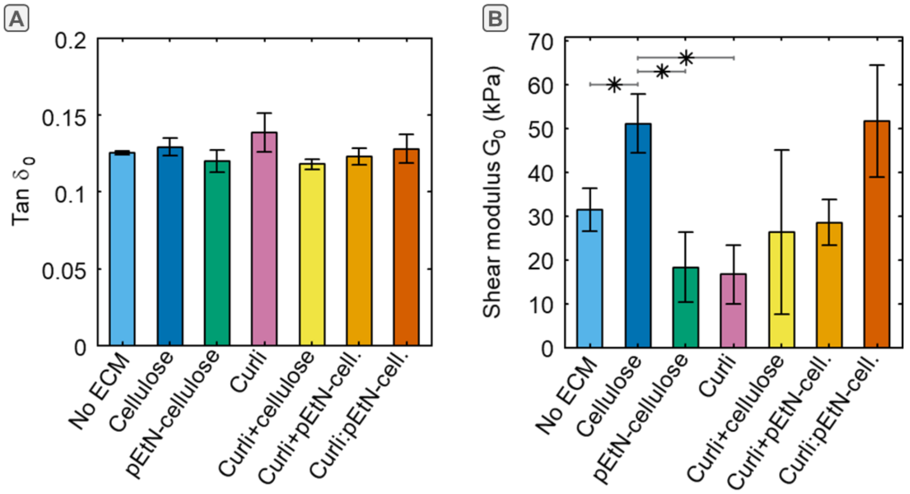

Figure S3. tan δ_0 and shear moduli G_0 calculated from G'_0 and G''_0: $\tan\delta_0 = \frac{G_0''}{G_0'}$ and $G_0 = \sqrt{G_0'^2 + G_0''^2}$

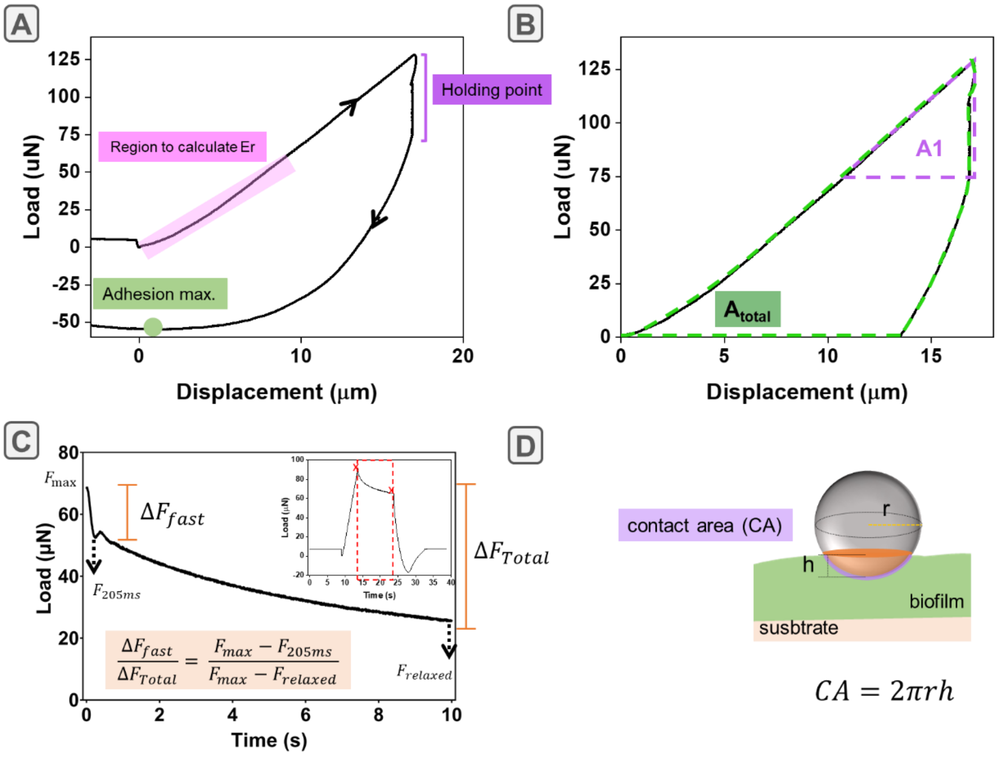

Figure S4. Microindentation of biofilms. (A) Representative load-displacement curve of a biofilm. A holding time of 10 s was set between loading and retraction of the tip. (B) Areas used for calculating the plasticity during the holding time: A1 is defined by the quasi-triangle formed by the loading curve and the load relaxation during the holding time and A_{total} is the area under the loading and detachment curves. (C) Analysis of biofilm relaxation behavior during the holding time. (D) Details of the contact area calculation used to determine the adhesion strength.

Table S5. Statistical significance for Fig. 3C; Biofilm reduced modulus E_r.

E_r	No ECM	Cellulose	pEtN-cellulose	Curli	Curli +cellulose	Curli +pEtN-cell.	Curli :pEtN-cell.
No ECM		ns	****	****	****	****	****
Cellulose	ns		****	****	****	****	****
pEtN-cellulose	****	****		****	****	****	ns
Curli	****	****	****		ns	****	ns
Curli+cellulose	****	****	****	ns		****	ns
Curli+pEtN-cell.	****	****	****	****	****		****
Curli:pEtN-cell.	****	****	ns	ns	ns	****	

Table S6. Statistical significance for Fig. 3E; $\Delta F_{fast}/\Delta F_{total}$.

$\Delta F_{fast}/\Delta F_{total}$	No ECM	Cellulose	pEtN-cellulose	Curli	Curli +cellulose	Curli +pEtN-cell.	Curli :pEtN-cell.
No ECM		***	****	****	****	ns	*
Cellulose	***		****	ns	ns	****	ns
pEtN-cellulose	****	****		****	**	****	**
Curli	****	ns	****		ns	****	ns
Curli+cellulose	****	ns	**	ns		****	ns
Curli+pEtN-cell.	ns	****	****	****	****		**
Curli:pEtN-cell.	*	ns	**	ns	**	**	

Table S7. Statistical significance for Fig. 3F; Plasticity at holding time ψ_h.

ψ_h	No ECM	Cellulose	pEtN-cellulose	Curli	Curli +cellulose	Curli +pEtN-cell.	Curli :pEtN-cell.
No ECM		ns	**	ns	ns	ns	ns
Cellulose	ns		*	ns	ns	ns	ns
pEtN-cellulose	**	*		***	***	***	ns
Curli	ns	ns	***		ns	ns	ns
Curli+cellulose	ns	ns	***	ns		ns	ns
Curli+pEtN-cell.	ns	ns	***	ns	ns		ns
Curli:pEtN-cell.	ns	ns	ns	ns	ns	ns	

Table S8. Statistical significance for Fig. 3G; Adhesion strength σ_{Adh}.

σ_{Adh}	No ECM	Cellulose	pEtN-cellulose	Curli	Curli +cellulose	Curli +pEtN-cell.	Curli :pEtN-cell.
No ECM		ns	ns	ns	***	ns	ns
Cellulose	ns		ns	ns	****	ns	*
pEtN-cellulose	ns	ns		ns	*	ns	ns
Curli	ns	ns	ns		*	ns	ns
Curli+cellulose	***	****	*	*		**	*
Curli+pEtN-cell.	ns	ns	ns	ns	**		ns
Curli:pEtN-cell.	ns	*	ns	ns	*	ns	

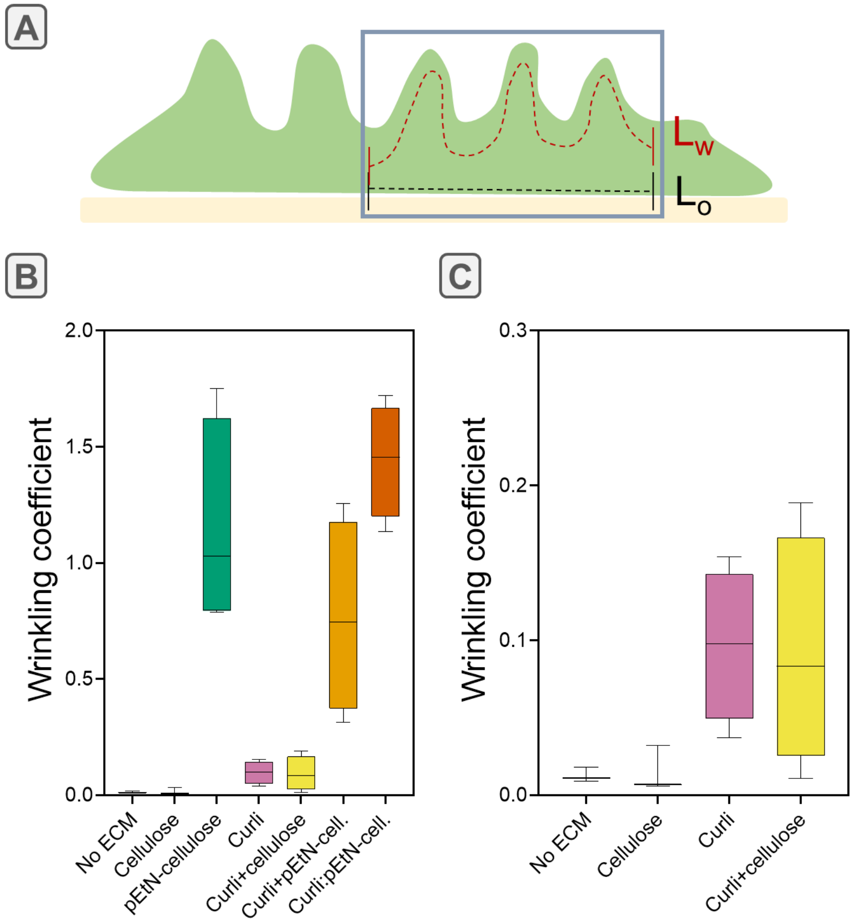

Figure S5. Quantification of biofilm wrinkling. (A) Determination of the wrinkling coefficient from biofilm cross sections. The wrinkling coefficient is defined as $(L_w-L_0)/L_0$, where L_w is the actual length of the path following the wrinkled biofilm and L_0 is the length of the straight line spanning the extremities of this path. (B) Wrinkling coefficient determined from biofilm cross-sections, as shown in Figure 4. The wrinkling coefficients were calculated for cross sections measured from three biofilms grown on different agar plates, which were seeded from the same bacterial suspension. (C) Zoom of B between 0 and 0.3.

Table S9. Statistical significance of the wrinkling coefficient in Fig. S5.

Wrinkling coefficient	No ECM	Cellulose	pEtN-cellulose	Curli	Curli +cellulose	Curli +pEtN-cell.	Curli :pEtN-cell.
No ECM		ns	***	ns	ns	*	****
Cellulose	ns		***	ns	ns	*	****
pEtN-cellulose	***	***		***	***	ns	****
Curli	ns	ns	***		ns	*	****
Curli+cellulose	ns	ns	***	ns		*	****
Curli+pEtN-cell.	*	*	ns	*	*		*
Curli:pEtN-cell.	****	****	ns	****	****	*	

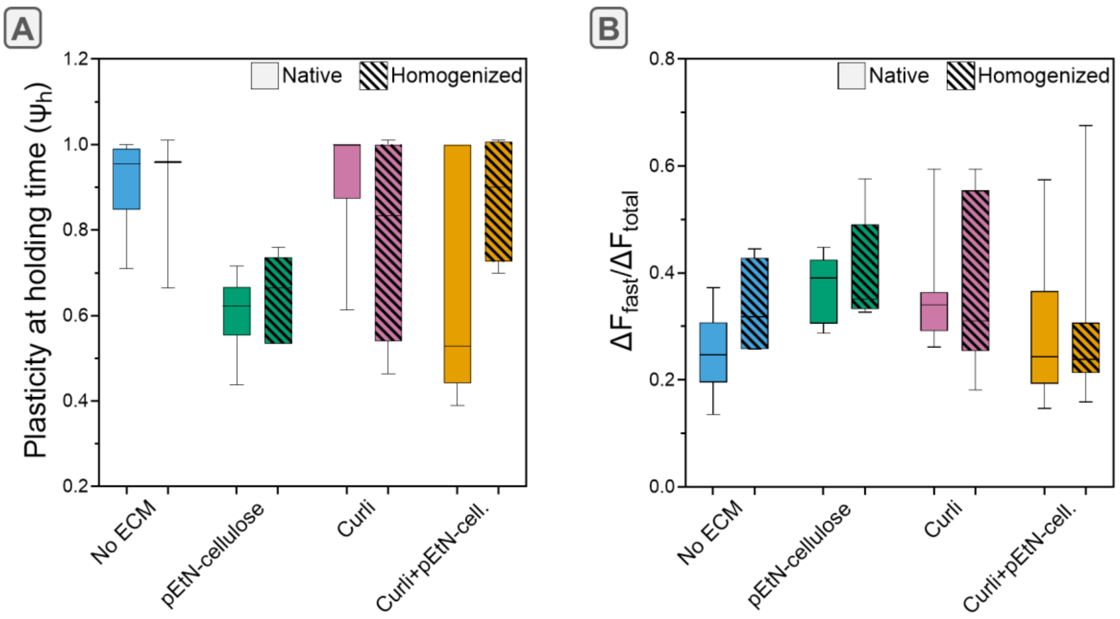

Figure S6. Influence of a homogenization step (mixing) on the plasticity (A) and the relaxation behaviour (B) of the biofilm.

Table S10. Statistical significance of the comparison between native vs. homogenized biofilms, shown in Fig. 5B and C.

Native vs. Homogenized	E_r	ψ_h	$\Delta F_{fast}/\Delta F_{total}$	σ_{Adh}
No ECM	ns	ns	ns	ns
pEtN-cellulose	ns	ns	ns	***
Curli+pEtN-cell.	ns	ns	ns	ns
Curli	ns	ns	*	*

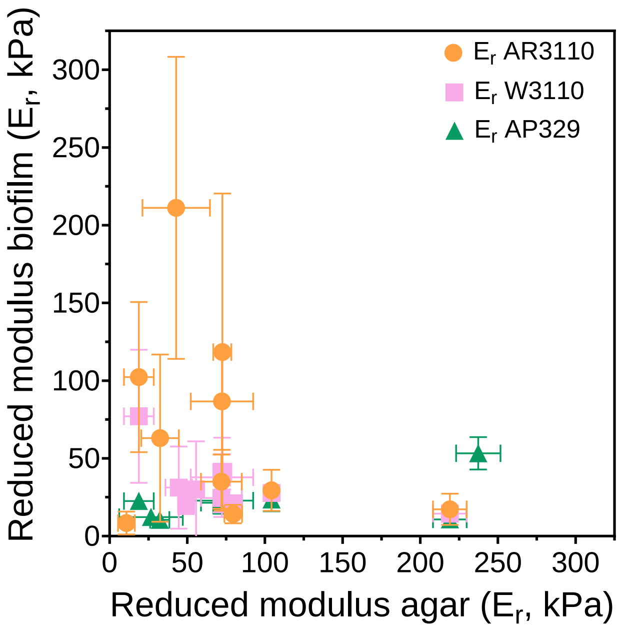

Figure S7. Comparative plot of the reduced elastic moduli of biofilms vs. agar plates.
